# Alteration of Vascular Responsiveness to Uridine Adenosine Tetraphosphate in Aortas Isolated from Male Diabetic Otsuka Long-Evans Tokushima Fatty Rats: The Involvement of Prostanoids

**DOI:** 10.3390/ijms18112378

**Published:** 2017-11-09

**Authors:** Takayuki Matsumoto, Shota Kobayashi, Makoto Ando, Maika Iguchi, Keisuke Takayanagi, Mihoka Kojima, Kumiko Taguchi, Tsuneo Kobayashi

**Affiliations:** Department of Physiology and Morphology, Institute of Medicinal Chemistry, Hoshi University, Shinagawa-ku, Tokyo 142-8501, Japan; sho.kobayan.1213@gmail.com (S.K.); redevil1126@gmail.com (M.A.); maika.iguchi@gmail.com (M.I.); k955tkyng@gmail.com (K.T.); cozymihomiho.0210@gmail.com (M.K.); k-taguchi@hoshi.ac.jp (K.T.)

**Keywords:** aorta, contraction, prostanoid, relaxation, type 2 diabetes, Up_4_A

## Abstract

We investigated whether responsiveness to dinucleotide uridine adenosine tetraphosphate (Up_4_A) was altered in aortas from type 2 diabetic Otsuka Long-Evans Tokushima Fatty (OLETF) rats compared with those from age-matched control Long-Evans Tokushima Otsuka (LETO) rats at the chronic stage of disease. In OLETF aortas, we observed the following: (1) Up_4_A-induced contractions were lower than those in the LETO aortas under basal conditions, (2) slight relaxation occurred due to Up_4_A, but this was not observed in phenylephrine-precontracted LETO aortas, (3) acetylcholine-induced relaxation was reduced (vs. LETO), and (4) prostanoid release (prostaglandin (PG)F_2α_, thromboxane (Tx)A_2_ metabolite, and PGE_2_) due to Up_4_A was decreased (vs. LETO). Endothelial denudation suppressed Up_4_A-induced contractions in the LETO group, but increased the contractions in the OLETF group. Under nitric oxide synthase (NOS) inhibition, Up_4_A induced contractions in phenylephrine-precontracted aortas; this effect was greater in the LETO group (vs. the OLETF group). The relaxation response induced by Up_4_A was unmasked by cyclooxygenase inhibitors, especially in the LETO group, but this effect was abolished by NOS inhibition. These results suggest that the relaxant component of the Up_4_A-mediated response was masked by prostanoids in the LETO aortas and that the LETO and OLETF rats presented different contributions of the endothelium to the response.

## 1. Introduction

Diabetes is one of the most common diseases occurring worldwide, yet it remains difficult to manage this disease and its associated complications [1]. The maintenance of vascular function is important for the prevention and improvement of diabetes-associated complications [2,3,4]. Although vascular dysfunction, including vascular tone abnormalities in response to various vasoactive substances, occurs systemically in diabetes, the mechanisms that underlie the initiation and development of this dysfunction are complex. Thus, for the urgent management of diabetes-associated complications, it is necessary to gain a comprehensive understanding of the differences among regions and disease stages (e.g., differences between macro- and microvasculature and among pre-, early, and chronic diabetes) in terms of the association between the signaling of vasoactive substances (including their generations), detectors (e.g., receptors), associated intracellular pathways, and cross-talk as well as the responses between vasoactive factors.

The endothelium is the largest endocrine organ and plays an important role in the regulation of vascular homeostasis [5,6,7,8,9,10]. Numerous vasoactive factors are released from the endothelium via various stimuli, and vascular tone is regulated through the release of various factors, including endothelium-derived relaxing factors (EDRFs) such as nitric oxide (NO), prostacyclin (PGI_2_), and endothelium-derived hyperpolarizing factor, as well as endothelium-derived contracting factors (EDCFs) such as endothelin-1 and vasoconstrictor prostanoids [9,10,11,12,13,14,15]. Several reports have demonstrated an imbalance between the production of EDRFs and EDCFs and their responses in diabetic arteries [9,14,16].

One of these endothelium-derived factors is uridine adenosine tetraphosphate (Up_4_A), which was first identified as an EDCF [17], but further investigation revealed it to be a vasoactive substance that induces many effects, including migration, proliferation, calcification, relaxation, and contraction, in endothelial and vascular smooth muscle cells [14,17,18,19,20,21,22,23,24,25,26,27,28,29,30,31,32,33,34,35,36,37,38]. Several reports have suggested regional heterogeneity in Up_4_A-mediated responses in vessels [14,24]. For example, Up_4_A led to contraction in mouse aortas [20], mouse renal arterioles [22], rat pulmonary arteries [19], and rat basilar, mesenteric, renal, and femoral arteries [25], whereas it led to relaxation in rat aortas [23], isolated perfused rat kidneys [31], and porcine coronary arteries [34,35]. Several reports have even suggested a pathogenetic role of Up_4_A. Moreover, circulating levels of Up_4_A were elevated in juvenile hypertensive patients [21], and Up_4_A injections led to an increase in mean arterial blood pressure in intact animals [17]. We previously observed that, compared with control uninephrectomized rats, Up_4_A-induced contraction was augmented in basilar, femoral, and renal arteries, but reduced in small mesenteric arteries of deoxycorticosterone acetate (DOCA)-salt hypertensive rats [24,25]. In addition to hypertension, we recently observed that (1) Up_4_A-induced contraction in renal arteries was increased in type 2 diabetic Goto-Kakizaki (GK) rats due to the activation of the cyclooxygenase (COX)/thromboxane (Tx) receptor pathway [28] and (2) Up_4_A-induced contraction in renal arteries was increased in type 2 diabetic Otsuka Long-Evans Tokushima Fatty (OLETF) rats with aging, and this contraction was suppressed via COX inhibition [27]. Zhou et al. [37] also reported that Up_4_A-induced contraction in mouse aortas was suppressed by COX inhibition. These results suggest that in some conditions there occurs cross-talk between Up_4_A and vasoconstrictor prostanoids in arteries, and these are important regulators of vascular function [8,11,12,14]; however, little is known regarding the relationship between COX-derived prostanoids and Up_4_A-induced responses in large arteries under diabetic conditions, especially long-term type 2 diabetes.

The OLETF rat was derived from spontaneous obesity in an outbred colony of Long-Evans rats [39,40]. OLETF rat and its control Long-Evans Tokushima Otsuka (LETO) lines were then developed by selective breeding [39,40]. The OLETF rat is a genetic animal model with cholecystokinin-1 receptor deficiency and is a well-established obese type 2 diabetic animal model [39,40,41]. This strain gradually develops hyperglycemia with obesity after birth, resembling human type 2 diabetes with obesity [39,40]. Several studies conducted by us and others have demonstrated altered vascular functions in various vessels [27,42,43,44,45,46,47,48,49,50]; however, no previous study has investigated whether responsiveness to Up_4_A in the aorta was altered in this model.

In this study, we hypothesized that alterations of vascular reactivity in response to Up_4_A in the aorta would be observed in OLETF rats at the chronic stage of disease. Using molecular and pharmacological approaches, we particularly investigated the relationships between Up_4_A-mediated responses and endothelium-derived factors in the diabetic aorta.

## 2. Results

### 2.1. General Parameters

As shown in Table 1, OLETF rats exhibited hyperglycemia compared with the age-matched control LETO rats. The systolic blood pressure (SBP) of the OLETF rats was higher than that of the LETO rats. The body weight of the OLETF rats was lower than that of the LETO rats.

### 2.2. Role of Endothelium in Up_4_A-Mediated Responses in the Aorta

**Figure 1:**
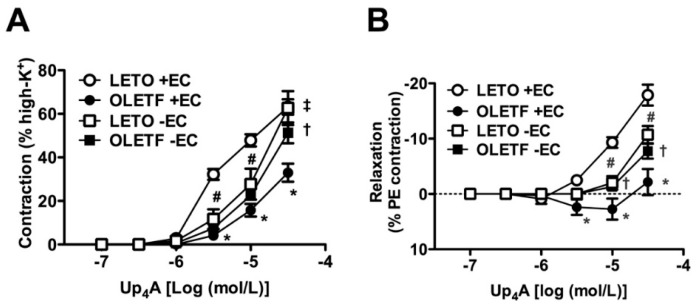
Contribution of the endothelium to cumulative applications of uridine adenosine tetraphosphate (Up_4_A) in the aortas of LETO and OLETF rats under basal conditions or after being precontracted with phenylephrine (PE). Concentration-response curves for Up_4_A in endothelium-intact (+EC) and -denuded (−EC) aortas under basal conditions (**A**) or precontracted with PE (10^−6^ mol/L). (**B**) The points show the means ± standard errors as percentages of the contraction normalized by high K^+^ (80 mmol/L) (**A**) or as percentages of the relaxation of the contraction induced by PE (10^−6^ mol/L) (**B**). *n* = 5–6. * *p* < 0.05, +EC LETO vs. +EC OLETF aortas. # *p* < 0.05, +EC LETO vs. −EC LETO aortas. † *p* < 0.05, +EC OLETF vs. −EC OLETF. ‡ *p* < 0.05, −EC LETO vs. −EC OLETF aortas. LETO, Long-Evans Tokushima Otsuka rats; OLETF, Otsuka Long-Evans Tokushima Fatty rats.

To determine the effects of Up_4_A on the aortic vascular tone and the relationship between such responses and the endothelium, Up_4_A was cumulatively applied to aortas with and without endothelium that had been isolated from OLETF and LETO rats under basal conditions (Figure 1A) or after being precontracted with phenylephrine (PE; 10^−6^ mol/L; Figure 1B). Under basal conditions, Up_4_A led to concentration-dependent contraction in both the OLETF and LETO groups. When the endothelium was intact, Up_4_A-induced aortic contractions were weaker in the OLETF group than in the LETO group. Endothelial denudation increased the Up_4_A-induced contractions in the aortas from the OLETF group, but reduced the contractions in those from the LETO group (Figure 1A). In the PE-precontracted aortas, a very small relaxant response to Up_4_A was observed in the OLETF group. By contrast, no relaxant response to Up_4_A was seen in the aortas from the LETO group (Figure 1B). Endothelial denudation eliminated the relaxant response and unmasked the contraction in the OLETF aortas. Conversely, in the LETO group, the contractile response induced by Up_4_A was reduced by endothelial denudation (Figure 1B).

### 2.3. Relaxation Induced by Acetylcholine and Sodium Nitroprusside in Endothelium-Intact Aortas

**Figure 2:**
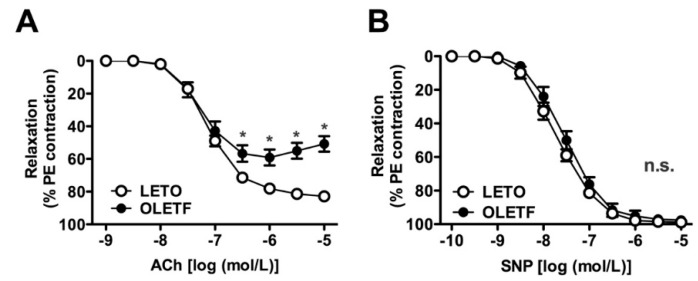
Concentration-response curves for acetylcholine (ACh) (**A**) or sodium nitroprusside (SNP) (**B**) in endothelium-intact aortas precontracted with phenylephrine (PE; 10^−6^ mol/L) isolated from LETO and OLETF rats. (**A**,**B**) The points show the means ± standard errors as percentages of the relaxation of the contraction induced by PE (10^−6^ mol/L). *n* = 5. * *p* < 0.05, LETO vs. OLETF. LETO, Long-Evans Tokushima Otsuka rats; OLETF, Otsuka Long-Evans Tokushima Fatty rats; n.s., not significant.

To investigate endothelial and smooth muscle functions, concentration-response curves of endothelium-intact aortas were plotted for acetylcholine (ACh) and sodium nitroprusside (SNP), which are well-known endothelium-dependent and -independent vasodilators, respectively (Figure 2). As shown in Figure 2A, ACh-induced relaxation was weaker in the aortas from the OLETF rats than in those from the LETO rats. However, SNP-induced relaxation did not differ between the two groups (Figure 2B).

### 2.4. Effects of Nitric Oxide Synthase (NOS) and COX Inhibitors on Up_4_A-Induced Aortic Relaxation

**Figure 3:**
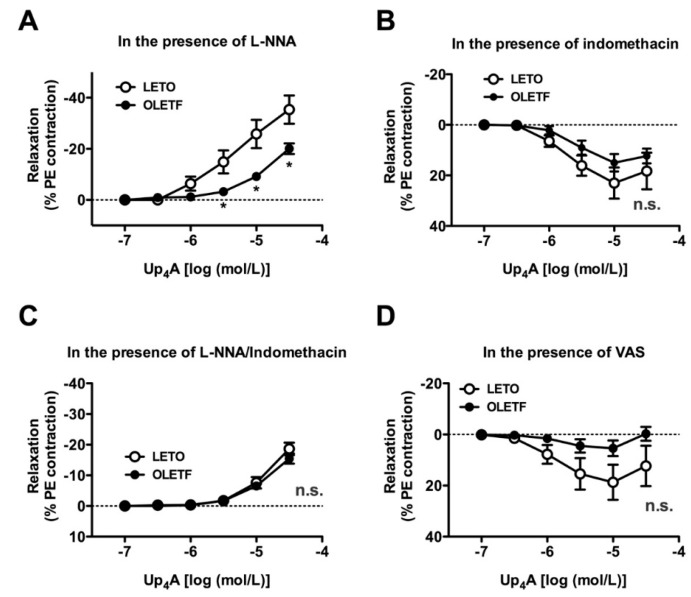

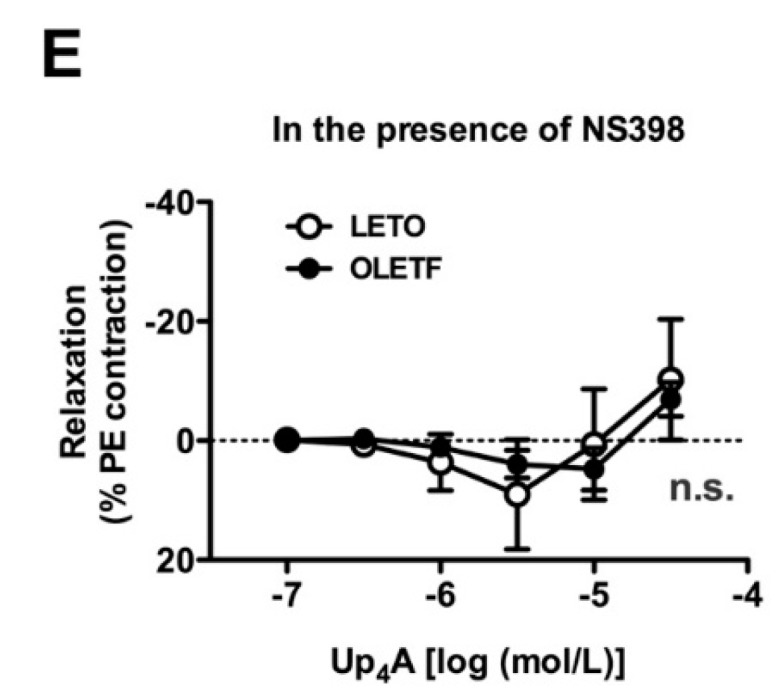
Effects of nitric oxide synthase (NOS) and cyclooxygenase (COX) inhibitors on the cumulative applications of uridine adenosine tetraphosphate (Up_4_A) in endothelium-intact aortas precontracted with phenylephrine (PE) from LETO and OLETF rats. Concentration-response curves for Up_4_A in endothelium-intact aortas precontracted with PE (10^−6^ mol/L) in the presence of a non-selective NOS inhibitor (L-NNA, 10^−4^ mol/L) (**A**), COX inhibitor (indomethacin, 10^−5^ mol/L) (**B**), L-NNA (10^−4^ mol/L) plus indomethacin (10^−5^ mol/L) (**C**), a selective COX1 inhibitor (VAS, 10^−4^ mol/L) (**D**), or a selective COX2 inhibitor (NS398, 10^−6^ mol/L) (**E**). The points show the means ± standard errors as percentages of the relaxation of the contraction induced by PE (10^−6^ mol/L (**B**,**D**,**E**) or 10^−7^ mol/L (**A**,**C**)). *n* = 6 or 8. * *p* < 0.05 corresponding LETO group. LETO, Long-Evans Tokushima Otsuka rats; OLETF, Otsuka Long-Evans Tokushima Fatty rats; n.s., not significant; L-NNA, N^G^-nitro-l-arginine; VAS, valeroyl salicylate.

Since (1) NO and COX-derived prostanoids play important roles in regulating vascular tone, (2) abnormalities in their signaling pathways contribute to vascular dysfunction [9,10,11,12,13,14], and (3) nitric oxide synthase (NOS) or COX signaling participates in Up_4_A-mediated responses in some vessels [20,23,27,28,37], we investigated whether Up_4_A-induced relaxations were associated with their activities. Under NOS inhibition by N^G^-nitro-L-arginine (L-NNA), Up_4_A induced concentration-dependent contractions in endothelium-intact PE-precontracted aortas; this effect was greater in the LETO group than in the OLETF group (Figure 3A). Surprisingly, relaxation responses induced by Up_4_A in the LETO group were unmasked in the presence of the non-selective COX inhibitor indomethacin (Figure 3B). Under NOS and COX inhibitions, similar contractile responses by Up_4_A were observed in both the OLETF and LETO groups (Figure 3C). In the LETO group, relaxation responses by Up_4_A were observed in aortas treated with each selective inhibitor of COX (COX1, Figure 3D; COX2, Figure 3E).

### 2.5. Effect of Up_4_A on Prostanoid Release in the Aorta

**Figure 4:**
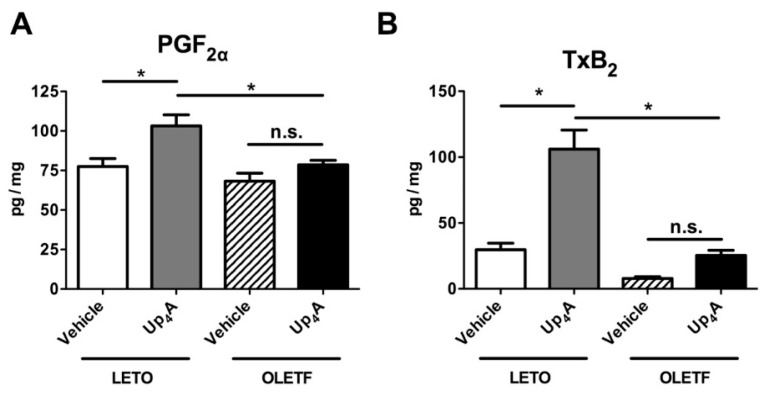

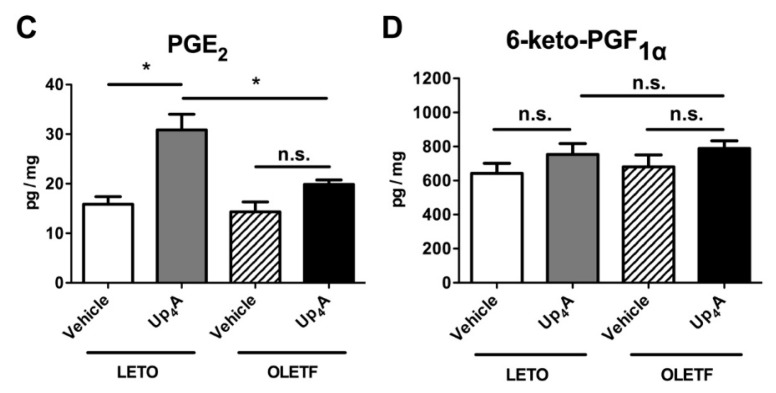
Release of the prostanoids prostaglandin (PG)F_2α_ (**A**), thromboxane (Tx)B_2_ (a stable metabolite of TxA_2_) (**B**), PGE_2_ (**C**), and 6-keto-PGF_1α_ (a stable metabolite of PGI_2_) (**D**), evoked by uridine adenosine tetraphosphate (Up_4_A; 3 × 10^−5^ mol/L) or vehicle (water) in aortic rings obtained from LETO (*n* = 7) and OLETF (*n* = 7) rats. The *y*-axis shows amounts of prostanoids expressed as pg/mg wet weight of aortic ring. Each column represents means ± standard error. * *p* < 0.05 vs. Up_4_A LETO. LETO, Long-Evans Tokushima Otsuka rats; OLETF, Otsuka Long-Evans Tokushima Fatty rats; n.s., not significant.

We next measured the production of prostanoids in the aorta stimulated by Up_4_A (Figure 4). In the LETO group, Up_4_A (3 × 10^−5^ mol/L) significantly increased the release of PGF_2α_ (Figure 4A), TxB_2_ (Figure 4B), and PGE_2_ (Figure 4C) compared with the vehicle-treated group. By contrast, no significant increases in the Up_4_A-induced production of these prostanoids were seen in the OLETF aortas. The release of the PGI_2_ metabolite (6-keto PGF_1α_) in the aorta was similar among the four groups (Figure 4D).

### 2.6. Expressions of COX Proteins in the Aorta

**Figure 5:**
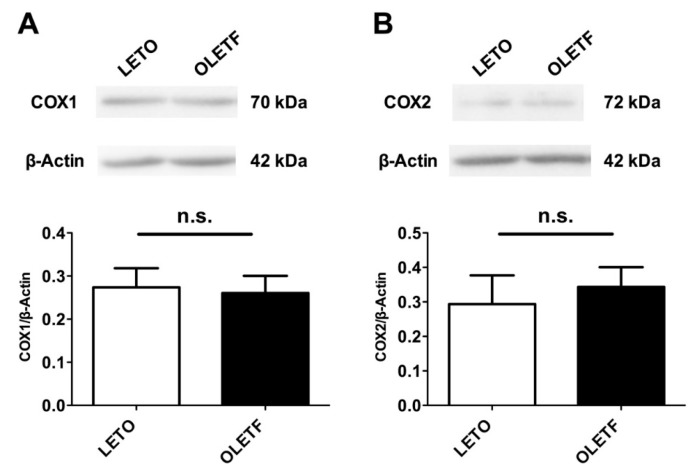
Protein expression of cyclooxygenase (COX)1 (**A**) and COX2 (**B**) in aortas obtained from LETO (*n* = 6) and OLETF (*n* = 6) rats. Each column represents means ± standard errors. LETO, Long-Evans Tokushima Otsuka rats; OLETF, Otsuka Long-Evans Tokushima fatty rats; n.s., not significant.

We next investigated whether the expression of COX proteins in the aorta differed between the two groups. An immunoblot analysis of aortas isolated from the LETO and OLETF rats was performed (Figure 5). No significant alterations of COX1 (Figure 5A) or COX2 (Figure 5B) were detected in either group of rats.

## 3. Discussion

In this study, we examined whether Up_4_A-induced responses were altered in aortas obtained from type 2 diabetic OLETF rats at the chronic stage of disease. The major findings of this study were that the responsiveness to Up_4_A in aortas differed between the OLETF and age-matched control LETO rats and that the contribution of the endothelium to Up_4_A-mediated aortic responses also differed between the two groups. We also observed that the relaxation response induced by Up_4_A was unmasked by the inhibition of COX, especially in the LETO group, and this relaxant response was abolished by NOS inhibition. Furthermore, the production of prostanoids induced by Up_4_A was higher in the aortas from the LETO group despite there being no differences between the two groups with respect to COX1 and COX2 expression in the aorta. Our results indicated that the endothelium and COX-derived prostanoids play roles in the Up_4_A-mediated responses in the aorta, and the contributions of the endothelium and COX-derived prostanoids to these responses differ over the long-term course of diabetes.

Up_4_A was originally identified as an EDCF [17] and a dinucleotide containing a purine and pyrimidine moiety [17]. Subsequent investigations have demonstrated that Up_4_A is a vasoactive substance with properties related to cell migration and proliferation in vascular smooth muscle cells, the development of calcification, the generation of reactive oxygen species, and the alteration of vascular tone [14,18,24,29]. After Up_4_A was first identified as an EDCF, early reports had stated that it could modulate vascular tone, including vasoconstrictions in perfused rat kidneys [17], rat pulmonary arteries [19], rat aortas [23], mouse aortas [20,38], and mouse renal arterioles [22] as well as vasodilation in rat aortas [23] and porcine coronary arteries [35]. In addition, several reports have demonstrated alterations of Up_4_A-induced responses in arteries. We previously observed heterogeneous effects on Up_4_A-mediated contraction among the various vascular beds in DOCA-salt hypertensive rats; compared with control uninephrectomized rats, Up_4_A-induced contraction in the DOCA-salt rats was (1) increased in the basilar, renal, and femoral arteries; (2) reduced in the small mesenteric artery; and (3) unchanged in the pulmonary artery and thoracic aorta [25,26]. Zhou et al. [35,36] detected that Up_4_A-mediated coronary vasodilation was impaired in a myocardial infarction model compared within sham-operated swine. Furthermore, we recently observed that Up_4_A-induced contractions in renal arteries increased in two different type 2 diabetic models: GK rats [28] and OLETF rats [27]. These findings suggest that responsiveness to Up_4_A varies among species, vessel types, and disease states. In the present study, we demonstrate for the first time that Up_4_A-induced aortic contraction increased in non-diabetic LETO rats compared to diabetic OLETF rats at the chronic stage of disease.

A novel, intriguing, and potentially important finding of this study was that the endothelium contributed to Up_4_A-mediated responses by opposing the suppressive effect in the aortas of OLETF rats and enhancing the effect against the contractile response in LETO rats; this was indicated by the increase of endothelial denudation and the reduction of Up_4_A-induced contraction in the aortas from the OLETF and LETO groups, respectively. Endothelial dysfunction is often seen in the arteries of type 2 diabetic patients. Indeed, endothelium-dependent relaxation was impaired in the aortas of type 2 diabetic animal models [42,43,51]. Accordingly, the present study determined that relaxation induced by the endothelium-dependent vasodilator ACh was impaired in the OLETF rats (compared with the LETO rats), whereas relaxation induced by the endothelium-independent vasodilator SNP was similar between the rat groups. These results suggest that there is endothelial dysfunction in the aortas of OLETF rats at the chronic stage. Our data indicate that the endothelium plays different roles in the regulation of vascular tone stimulated by each ligand (i.e., ACh and Up_4_A). Indeed, different extents of relaxation induced by each endothelium-dependent vasodilator have been observed in arteries from diabetic cases and controls [47,52,53]. Furthermore, Zhou et al. [36] recently reported that Up_4_A-induced coronary vasodilation was preserved in swine with metabolic derangement compared within normal swine, despite the impaired endothelium-dependent relaxation induced by bradykinin. However, these alterations may be surrogate and/or compensatory phenomena resulting from endothelial dysfunction; future investigation of the role of Up_4_A-mediated responses is needed.

In the physiological state, there is a balance among the endothelium-derived factors, including the EDRFs and EDCFs, and these factors regulate vascular homeostasis under stimulation by blood flow and various factors (e.g., neurotransmitters, hormones, and cytokines) [7,9,10,11,12,14]. Indeed, our present findings and previous reports have suggested that Up_4_A-induced vasomotion was regulated by the endothelium [23,28,35,38]. COX-derived prostanoids are not only EDCFs but also key regulators in the development of diabetes-associated vascular dysfunction [9,11,12,14,27,44,45,46,47,48,54]. Moreover, we and others have observed the interaction between COX-derived prostanoids and Up_4_A-mediated responses in the vasculature [20,27,28,37,38]. In the present study, the relaxant response induced by Up_4_A was unmasked by the suppression of COX, especially in the LETO group. In comparison, these modulatory effects on the Up_4_A-induced responses were minor in the OLETF group. To confirm the difference in vascular function, we explored the release of prostanoids following Up_4_A stimulation and determined that the levels of prostanoid release stimulated by Up_4_A differed between the aortas from the LETO and OLETF rats, with higher releases of PGF_2α_, TxA_2_ metabolites, and PGE_2_ seen in the LETO aortas. These data strongly supported the functional data because these prostanoids can induce vasocontraction [44,55]. Unlike the release of these prostanoids, the release of PGI_2_ (as measured from its metabolites) did not differ between the groups. The expression of COXs has been associated with nucleotide-induced vascular responses [27,28,37,54,56]. In the present study, the protein expressions of COX1 and COX2 in the aorta did not differ between the two groups of rats. These results imply that the regulation of the activity of each prostanoid synthase in the aorta may differ between LETO and OLETF rats. This idea is supported by a range of evidence suggesting that the regulation of the activity of each prostanoid synthase differs in (patho)physiological states [57,58,59].

In the aorta, NO is a major EDRF [51,53,60]. Indeed, Up_4_A-induced contraction is enhanced by NO synthase inhibition [23,26,28]. Furthermore, counteractions between NO and COX-derived prostanoids for vascular function have been observed in vessels in some conditions such as diabetes [9,10,11,12,13,14,47]. In the present study, we found that (1) the increased contractile response induced by Up_4_A under NOS inhibition was seen in the LETO group compared with the OLETF group; (2) relaxant responses induced by Up_4_A were observed in both groups under COX inhibition; and (3) such relaxant responses induced by Up_4_A under COX inhibition were abolished by co-treatment with NOS inhibitors. When this relevant evidence and our findings are taken into account, we speculate that in the LETO aorta, the contribution of prostanoids is stronger than that of NO, whereas both components are present in lesser amounts in OLETF aortas than in LETO aortas following Up_4_A stimulation.

The present study has some limitations. Because Up_4_A is a dinucleotide containing a pyrimidine and purine moiety, it is considered to be able to bind to purinoceptors [61]. Indeed, several reports have suggested that Up_4_A-induced vasomotor activities were suppressed by some purinoceptor antagonists [19,26,33,34,37]. In mouse aorta, Zhou et al. [37] found that Up_4_A-mediated contraction was due to TxA_2_ production, which partly required the activation of P2X_1_ receptor via an endothelium-dependent mechanism. Moreover, in the rat aorta, Linder et al. [23] found that Up_4_A-induced contraction was modulated by NO and mediated by activations of P1 and P2X receptors, and suggested the possible involvement of P2Y receptors in the Up_4_A-induced contraction. So far, we cannot state which receptor(s) primarily affect aortic responsiveness to Up_4_A because purinoceptor signaling is complex and there may be specific dinucleotide receptors [62,63,64,65,66]. However, further investigation of the relationships among receptors, NO, prostanoids, and functions after Up_4_A stimulation in the models is required.

In conclusion, our findings suggest that responsiveness to Up_4_A differs between the aortas of long-term type 2 diabetes OLETF rats and those of age-matched control LETO rats, and these differences may be due to the contribution of endothelium and prostanoid signaling. Investigating the signal transduction and regulation of vascular tone occurring via Up_4_A stimulation may be of significance in providing a comprehensive understanding of the pathogenesis of diabetes-associated vascular complications.

## 4. Materials and Methods

### 4.1. Animals and Procedures

All animal experiments were conducted according to the Guiding Principles for the Care and Use of Laboratory Animals from the Committee for the Care and Use of Laboratory Animals of Hoshi University, which is accredited by the Japan Ministry of Education, Culture, Sports, Science, and Technology. Four-week-old male (OLETF (*n* = 20) and LETO (*n* = 21)) rats were obtained from Hoshino Laboratory Animals, Inc. (Ibaraki, Japan). All the animals were maintained in an environmentally controlled room under a 12:12-h light:dark cycle and allowed free access to a standard laboratory animal chow (MF; Oriental Yeast Co., Ltd., Tokyo, Japan) and drinking water. SBP was measured using the tail-cuff method at least one week before sacrifice, as reported previously [46,47,48]. At the time of sacrifice, non-fasted blood glucose was measured under anesthesia by using a commercially available glucose meter (OneTouch Ultra, LifeScan, a Johnson & Johnson Company, Milpitas, CA, USA) [27,28].

### 4.2. Preparation of Rat Aortic Rings and Evaluation of Vascular Function

Vascular function was measured as reported previously [27,28,67]. In all experiments, non-fasted rats were nasally anesthetized with isoflurane (initially at 5% and then maintained at 2.5%) and euthanized by thoracotomy and exsanguination. After euthanasia, the thoracic aorta was carefully and rapidly isolated and placed in an ice-chilled, oxygenated, modified Krebs-Henseleit solution (KHS; consisting (in mM) of 118.0 NaCl, 4.7 KCl, 25.0 NaHCO_3_, 1.8 CaCl_2_, 1.2 NaH_2_PO_4_, 1.2 MgSO_4_, and 11.0 glucose). The aorta was separated from the surrounding connective tissue and cut into rings with a length of 2 mm. The rings were stretched until an optimal resting tension of 2.75 g was enforced, and changes in their tension were analyzed using a force-displacement transducer linked to a PowerLab recording system (AD Instruments, Australia). After equilibration (for ca. 45 min), arterial integrity was checked by contracting the rings with high K^+^ (80 mmol/L) and subsequently with PE (10^−6^ mol/L), followed by relaxation with ACh (10^−6^ or 10^−5^ mol/L). After washing and restabilization, Up_4_A (10^−7^–3 × 10^−5^ mol/L) was added cumulatively to the bath until a maximal response was achieved. To investigate relaxation response, aortic rings were precontracted with PE (10^−7^ or 10^−6^ mol/L). Once the PE-induced contraction had stabilized, Up_4_A (10^−7^–3 × 10^−5^ mol/L [27,28]), ACh (10^−9^–10^−5^ mol/L [27,28]), or SNP (10^−10^–10^−5^ mol/L [27,28]) was added cumulatively. To investigate the effects of NOS or COX inhibitors on the Up_4_A-induced responses, a ring was incubated for 30 min in the appropriate drug-containing medium, namely, 10^−4^ mol/L of L-NNA (a non-selective NOS inhibitor [26,28,54]), 10^−5^ mol/L indomethacin (a non-selective COX inhibitor [28,54]), 10^−4^ mol/L of valeroyl salicylate (VAS; a selective COX1 inhibitor [28,54]), 10^−6^ mol/L of NS398 (a selective COX2 inhibitor [28,54]), or L-NNA (10^−4^ mol/L) plus indomethacin (10^−5^ mol/L) before the addition of PE. These concentrations of drugs were chosen based on previous studies [26,27,28,54]. When required, removal of the endothelium from the aortic segments was achieved by gently rubbing the lumen side of the vessels with a pipette tip.

### 4.3. Measurement of Prostanoid Release

Prostanoid release was measured essentially as described in our previous papers [45,46,47,48,54]. In brief, aortic rings with a length of 4 mm were incubated in 1.0 mL of KHS at 37 °C. The rings were then rapidly transferred to siliconized tubes containing 0.5 mL of KHS in the presence of Up_4_A (3 × 10^−5^ mol/L) or vehicle (water) at 37 °C for 5 min. After the aortic rings were removed and weighed, the tubes were freeze-clamped in liquid nitrogen and stored at −80 °C for later analysis. The prostanoids (or stable metabolites), including PGF_2α_, TxB_2_, (a stable metabolite of TxA_2_), PGE_2_, and 6-keto PGF_1α_ (a stable metabolite of PGI_2_), were measured using a commercially available enzyme immunoassay (EIA) kit (Cayman Chemical, Ann Arbor, MI, USA), as described in the manufacturer’s procedure booklet. The amounts of PGs released are expressed as pg/mg wet weight of aortic ring.

### 4.4. Western Blotting

Protein expression was quantified using immunoblotting, as reported previously [27,28]. Aortic tissues from the rats were rapidly isolated and frozen in liquid nitrogen. Aortic protein extracts (30 μg) were subjected to 10% sodium dodecyl sulfate-polyacrylamide gel electrophoresis and transferred to polyvinylidene difluoride membranes. Blots were incubated with anti-COX1 (~70 kDa; 1:1000, Cayman Chemical (#160109, Ann Arbor, MI, USA)), anti-COX2 (~72 kDa; 1:1000, Cayman Chemical (#160126, Ann Arbor, MI, USA)), and anti-β-actin (#A5316, 42 kDa; 1:5000) antibodies, and detection was achieved using horseradish peroxidase-conjugated immunoglobulin G followed by enhanced chemiluminescence. To normalize the data, we used β-actin as a housekeeping protein. The bands were analyzed using CS Analyzer 3.0 software (ATTO, Tokyo, Japan).

### 4.5. Statistical Analysis

The results are expressed as means ± standard error, with *n* representing the number of animals used in the experiments. Statistical evaluations between two groups were performed using Student’s *t*-test, and one-way analysis of variance (ANOVA) followed by Tukey’s testing was used for comparisons of three or more groups. The concentration-response curves were statistically evaluated using two-way repeated measures ANOVA, followed by Bonferroni post hoc testing. These statistical analyses were made using Graph Pad Prism (v. 5.0; GraphPad Software Inc., San Diego, CA, USA). Differences were considered significant when *p* < 0.05.

## Figures and Tables

**Table 1:** General parameters.

Group	Body Weight (g)	Blood Glucose (mg/dL)	Systolic Blood Pressure (mmHg)
LETO	574.7 ± 10.7 (21)	108.5 ± 3.6 (21)	123 ± 4 (9)
OLETF	521.0 ± 14.7 (20) *	380.9 ± 28.8 (20) *	146 ± 6 (8) *

Values are presented as the mean ± standard error. The number of experiments is shown within parentheses. * *p* < 0.05, LETO vs. OLETF. LETO, Long-Evans Tokushima Otsuka rats; OLETF, Otsuka Long-Evans Tokushima Fatty rats.

## Abbreviations

ACh	Acetylcholine
ANOVA	Analysis of variance
COX	Cyclooxygenase
DOCA	Deoxycorticosterone acetate
EDCF	Endothelium-derived contracting factor
EDRF	Endothelium-derived relaxing factor
GK	Goto-Kakizaki
KHS	Krebs-Henseleit solution
LETO	Long-Evans Tokushima Otsuka
L-NNA	N^G^-nitro-l-arginine
NO	Nitric oxide
NOS	Nitric oxide synthase
OLETF	Otsuka Long-Evans Tokushima Fatty
PE	Phenylephrine
PG	Prostaglandin
SBP	Systolic blood pressure
SNP	Sodium nitroprusside
TP	Thromboxane-prostanoid receptor
Tx	Thromboxane
Up_4_A	Uridine adenosine tetraphosphate
VAS	Valeroyl salicylate
